# Successful treatment of pulmonary lymphatic perfusion syndrome with lymphatic intervention embolization: a case report

**DOI:** 10.3389/fmed.2025.1696594

**Published:** 2025-12-02

**Authors:** Qunxiang Liu, JiaQi Wang, Yingjie Chen, Wei Qin, Ziyang Zhu

**Affiliations:** Department of Pulmonary and Critical Care Medicine, The Sixth Hospital of Wuhan, Affiliated Hospital of Jianghan University, Wuhan, China

**Keywords:** pulmonary lymphatic perfusion syndrome (PLPS), chylous pericardial effusion, chyloptysis/milky sputum, thoracic duct embolization, lymphatic intervention

## Abstract

Pulmonary Lymphatic Perfusion Syndrome (PLPS) is a rare condition characterized by abnormal lymphatic drainage into the lungs, leading to symptoms such as chyloptysis and chylous effusions. We report a case of a 54-year-old male with PLPS who presented with persistent cough, milky sputum, and pericardial effusion. Following initial misdiagnosis and ineffective empirical treatment for a presumed pulmonary infection, a definitive diagnosis of PLPS was established. The patient underwent successful percutaneous thoracic duct embolization, resulting in complete resolution of symptoms and imaging findings at follow-up. This case highlights the importance of considering PLPS in patients with unexplained chylous effusions and the efficacy of lymphatic intervention as a treatment option.

## Introduction

Pulmonary Lymphatic Perfusion Syndrome (PLPS) is a rare, challenging disorder characterized by abnormal lymphatic flow from the thoracic duct into the lungs and surrounding structures (1). This aberrant perfusion can lead to severe clinical manifestations, including chylothorax, chylopericardium, and plastic bronchitis (1). Although often congenital, PLPS symptoms may be unmasked by secondary triggers like trauma or infection (2). Diagnosing PLPS remains difficult due to its non-specific presentation mimicking other conditions (3). While advanced imaging, particularly dynamic contrast-enhanced magnetic resonance lymphangiography (DCMRL), is crucial for identification (1), effective treatment remains a hurdle. Conservative measures often fail (4), creating a need for more definitive interventions. Percutaneous lymphatic embolization has emerged as a highly effective, minimally invasive treatment to occlude these abnormal pathways. Here, we report a 54-year-old male with PLPS who, after initial misdiagnosis and failed conservative therapy, was successfully treated with percutaneous thoracic duct embolization (5).

## Case presentation

**FIGURE 1:**
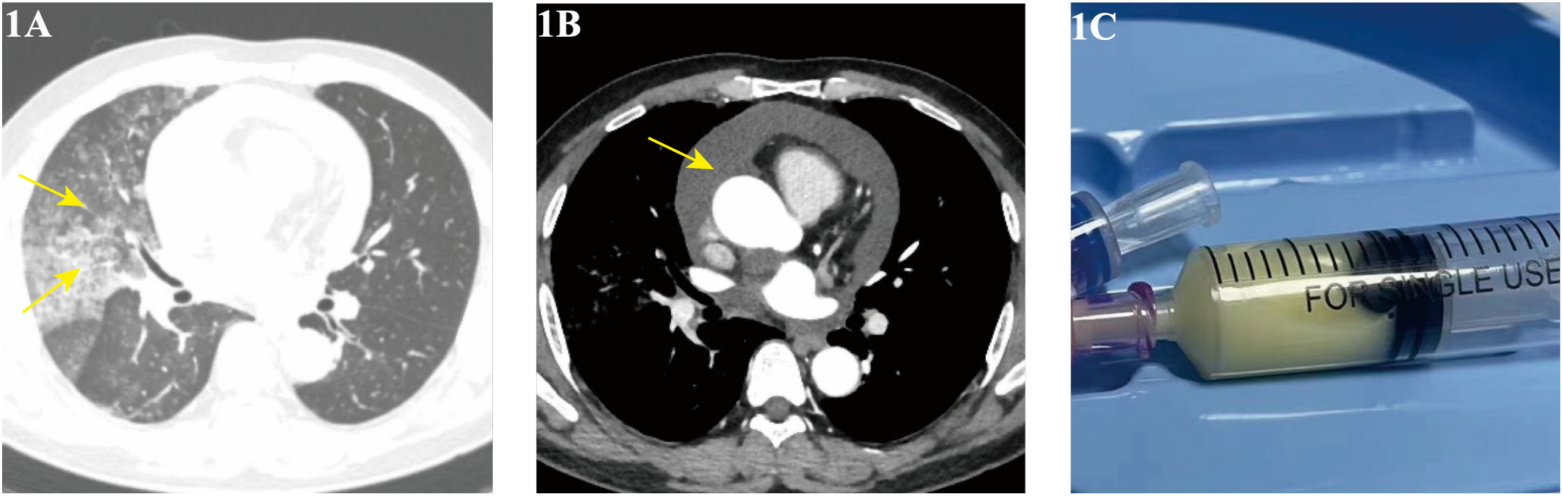
**(A,B)** Chest CT (August 29, 2023) showing right lung infiltrates and pericardial effusion; **(C)** pericardiocentesis yielding chylous fluid.

A 54-year-old male with a history of chronic bronchitis and hepatitis B presented with an 11-month history of intermittent cough, milky sputum, and fever, with a recent recurrence 20 days prior to admission on August 28, 2023. Initial treatment at another hospital for suspected lung infection and pericardial effusion (with antibiotics and pericardial drainage) provided partial relief of fever and dyspnea but no improvement in cough or sputum. Upon admission, physical examination revealed hypertension (147/108 mmHg) and coarse breath sounds with wet rales in the right lung. Chest CT showed a right lung infectious lesion (Figure 1A) and pericardial effusion (Figure 1B), while bronchoscopy revealed milky white sputum, and pericardial fluid (Figure 1C) analysis confirmed chylous effusion (high triglyceride levels, predominantly lymphocytes).

**FIGURE 2:**
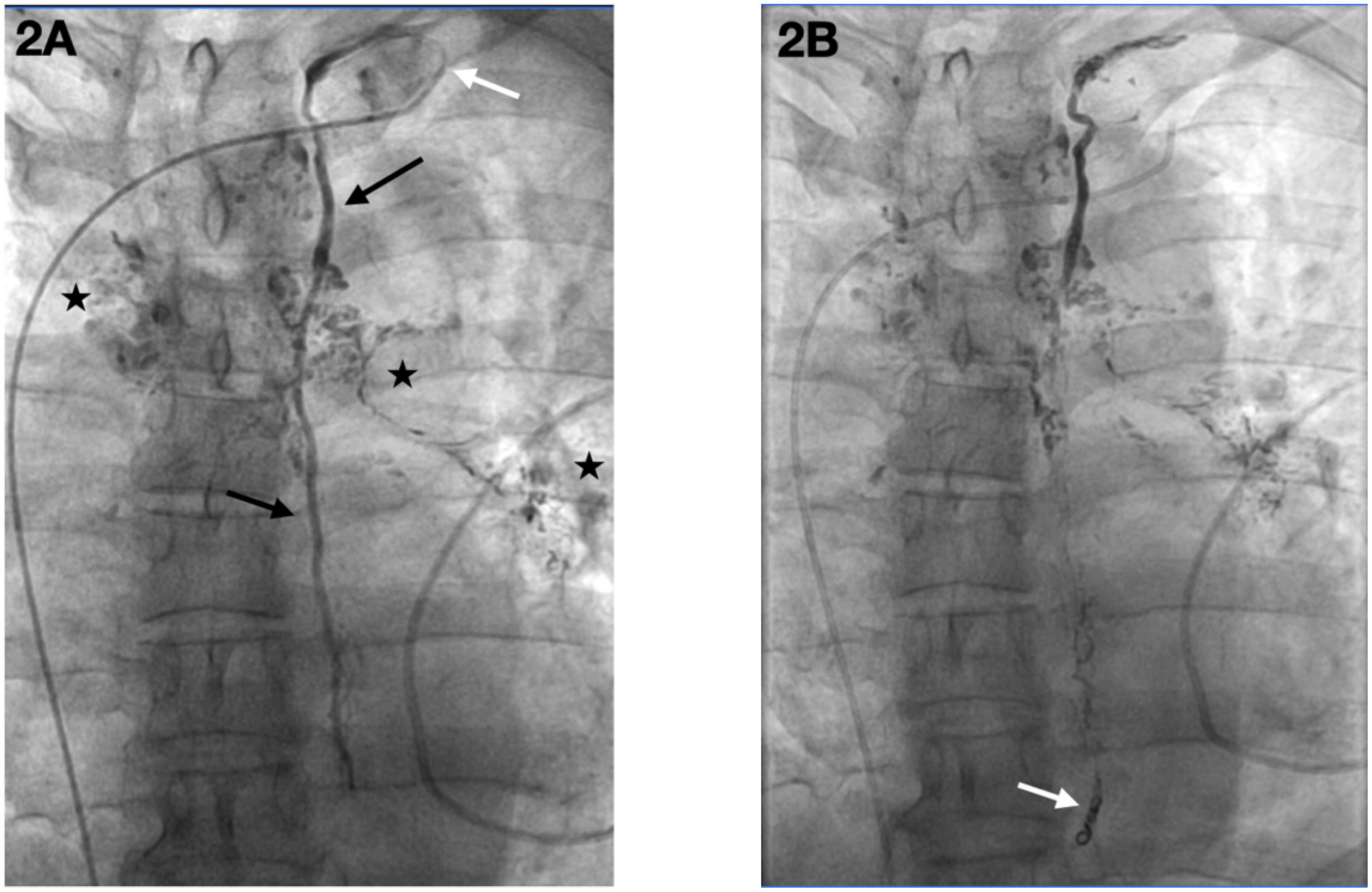
Lymphangiography and thoracic duct embolization. **(A)** Pre-embolization lymphangiography via a microcatheter (white arrow) in the thoracic duct shows the main duct trunk (black arrows) and an active fistula with contrast extravasation into the right lung parenchyma and pericardial cavity (asterisks). **(B)** Post-embolization DSA confirms successful occlusion of the thoracic duct, with the coil and glue cast visible (white arrow) and no further contrast leakage.

On September 2, 2023, the patient underwent a multi-step lymphatic interventional procedure. Initially, ultrasound-guided intranodal lymphangiography was performed via puncture of an inguinal lymph node, followed by the slow injection of iodized oil (Lipiodol). Imaging revealed lymphatic drainage into the left venous angle and its confluence with the subclavian vein. Concurrently, contrast extravasation was observed from the cisternal segment of the thoracic duct, with leakage extending toward the right lung and pericardium, confirming the diagnosis of PLPS (Figure 2A, Supplementary Video 1). Subsequently, the femoral vein was punctured, and a 5F vascular sheath was inserted. A 5F VERT catheter, advanced over a 0.035-inch guidewire, was navigated to the left subclavian vein to locate the lymphatic orifice. Upon identification of the orifice, a 1.98F microcatheter, supported by 0.014-inch and 0.018-inch microwires, was successfully advanced into the thoracic duct. Embolization of the distal thoracic duct was achieved using 3 × 3 microcoils (Supplementary Video 2), followed by the rapid injection of surgical glue (n-BCA) into the duct (Supplementary Video 3). This combined technique uses the coils to create a physical scaffold, slowing chyle flow and preventing glue migration, while the glue permeates the coil mesh to create a durable, impermeable seal. The microcatheter was promptly withdrawn post-injection. Final Digital Subtraction Angiography (DSA) confirmed satisfactory deposition of the surgical glue within the thoracic duct and demonstrated complete occlusion of the fistula, with no residual contrast extravasation (Figure 2B and Supplementary Video 4). Post-procedure, the patient was placed on a low-fat diet.

**FIGURE 3:**
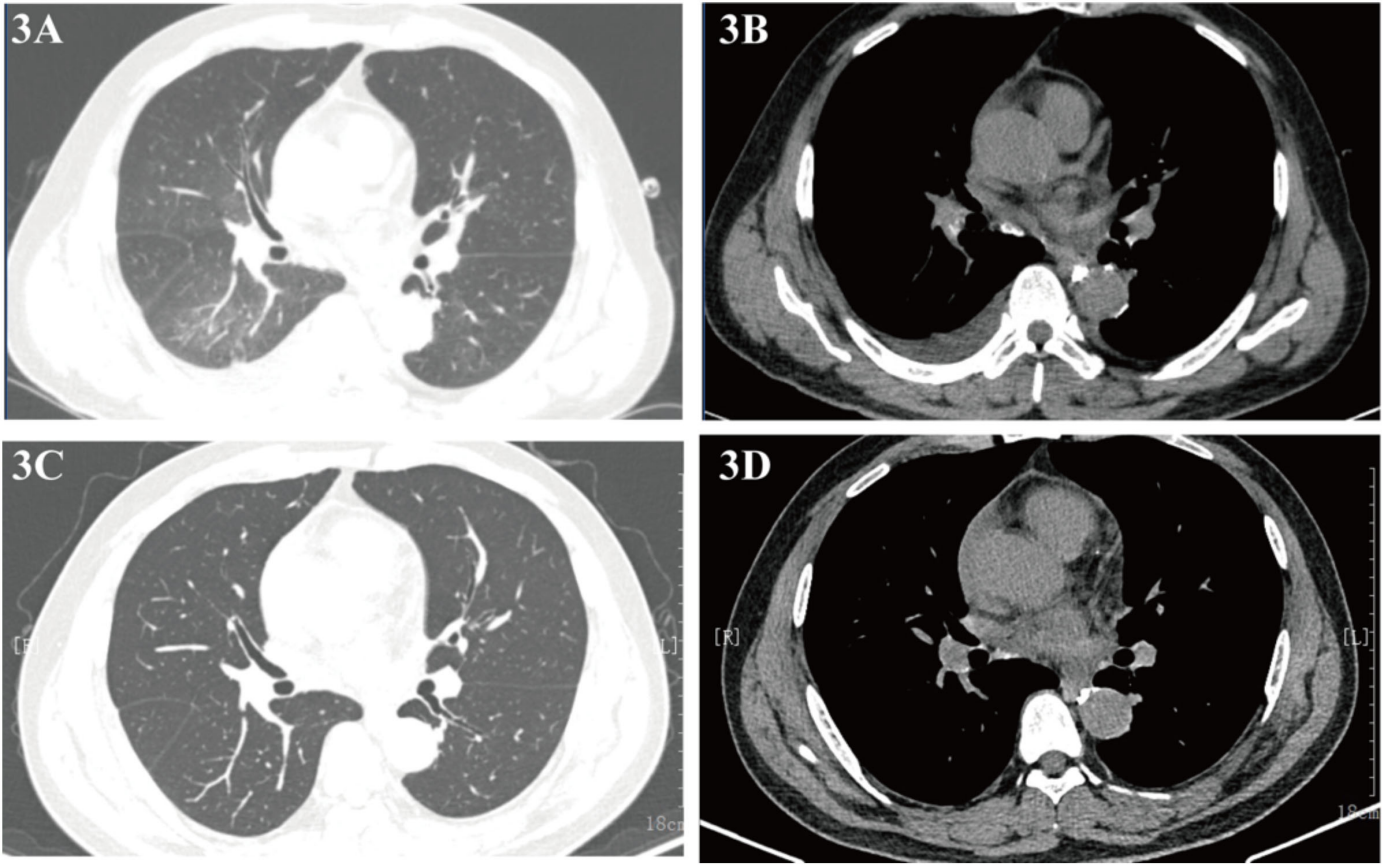
**(A,B)** Chest CT (September 4, 2023) showing reduced infiltrates and effusion; **(C,D)** follow-up CT (October 25, 2023) confirming resolution.

Follow-up imaging on September 4 and 8, 2023, revealed reduced lung infection and minimal pericardial effusion (Figures 3A, B), with complete resolution of both pericardial effusion and lung lesions observed by October 25, 2023 (Figures 3C, D), and no recurrence of symptoms. As of the latest follow-up, one year later, the patient reports no discomfort.

## Discussion

Pulmonary Lymphatic Perfusion Syndrome (PLPS) is a rare and complex group of lymphatic disorders characterized by the abnormal flow of chyle from the thoracic duct (TD) or other central lymphatic channels into the pulmonary parenchyma, airways, mediastinum, pericardium, or pleural space (6). This case report describes the successful management of a 54-year-old male presenting with the exceedingly rare combination of chyloptysis and chylous pericardial effusion secondary to PLPS, effectively treated with percutaneous thoracic duct embolization (TDE). The initial misdiagnosis as a pulmonary infection with reactive pericardial effusion underscores the diagnostic challenges posed by PLPS, where non-specific symptoms often mimic more common cardiorespiratory conditions (7).

The simultaneous occurrence of chyloptysis (indicative of lymphatic leakage into the bronchial tree or lung parenchyma) and chylopericardium (chyle accumulation in the pericardial sac) is an exceptionally uncommon clinical scenario. While primary or idiopathic chylopericardium is itself a rarity, with historical estimates of around 100 reported cases over decades (8–10), and chyloptysis is considered even rarer, with fewer than 20 cases documented by a 2018 review (2, 11), their co-existence strongly implicates a singular, central lymphatic pathology affecting major lymphatic pathways. PLPS provides a coherent pathophysiological explanation for such multi-compartmental chylous leakage (8). In this patient, lymphangiography confirmed active leakage from the thoracic duct into both the right lung and the pericardium (Figure 2A), directly supporting PLPS as the unifying cause of his symptoms. This abnormal perfusion is thought to arise from congenital anatomical lymphatic variants (1), where triggers such as infection or increased central venous pressure can unmask the underlying defect by increasing lymphatic flow or pressure (4).

The diagnostic pathway in this case highlights critical aspects of evaluating suspected lymphatic disorders. Confirmation of the chylous nature of the expectorated sputum and pericardial fluid was paramount, achieved through bronchoscopy revealing milky sputum and pericardial fluid analysis showing high triglyceride levels and a predominance of lymphocytes (8, 12). Although dynamic contrast-enhanced magnetic resonance lymphangiography (DCMRL) is the preferred non-invasive examination for evaluating PLPS, its diagnostic utility can be limited by a variety of technical and patient-specific factors. These include motion artifact, concomitant venous enhancement that may obscure lymphatic channels, and insufficient signal-to-noise ratio, particularly when the central lymphatic ducts are not pathologically dilated (13, 14), In our patient, the attempted DCMRL was non-diagnostic due to poor signal quality, a recognized challenge of the technique. We therefore proceeded with conventional catheter lymphangiography, which remains an essential tool for definitive diagnosis and interventional planning when advanced imaging is inconclusive or unavailable (15). The ability of lymphangiography to pinpoint the direct source of bilateral leakage (8) was crucial for both confirming PLPS and planning the subsequent interventional procedure (16).

Management of PLPS and associated chylous effusions has evolved significantly. Conservative measures, including a low-fat diet (8) (often supplemented with medium-chain triglycerides) and somatostatin analogs (17) (e.g., octreotide), aim to reduce chyle production and flow (2). However, these approaches often have limited success, particularly in cases of high-output or persistent leaks (18), or when complex anatomical abnormalities like PLPS are present, as was the experience with this patient prior to definitive intervention. The failure rates for conservative management, especially in non-traumatic contexts, can exceed 50–60% (17).

In contrast, percutaneous lymphatic interventions, particularly TDE or selective lymphatic embolization (SLE), have emerged as highly effective and minimally invasive treatments (16, 19, 20). TDE aims to occlude the thoracic duct at or near the point of leakage, thereby preventing further chylous extravasation. In this patient, embolization of the thoracic duct fistula using microcoils and surgical glue resulted in the rapid and complete resolution of both chyloptysis and pericardial effusion, with sustained clinical improvement at one-year follow-up. The combined embolization technique using microcoils and n-BCA glue is an established strategy to ensure durable fistula occlusion. The coils provide a stable 3D scaffold that slows flow and prevents distal migration of the liquid embolic, while the n-BCA glue permeates this framework to create a completely impermeable seal, thereby minimizing the risk of recanalization. This outcome is consistent with a growing body of evidence supporting TDE as a first-line invasive treatment for non-traumatic chylous effusions and specific manifestations of PLPS (16), such as plastic bronchitis (21) and chylothorax. Systematic reviews and institutional series report technical success rates for TDE in traumatic chylothorax as high as 70–90% (22), and with the advent of advanced imaging-guided algorithms, success in complex non-traumatic chylothorax, including PLPS, can reach over 90% in specialized centers (6). Compared to traditional thoracic duct ligation (TDL), TDE offers significant advantages in terms of lower procedural morbidity, shorter recovery times, and reduced invasiveness. While TDL remains a highly effective treatment, it typically requires open thoracic surgery and is associated with the risks of a major operation. The current treatment paradigm for refractory chyle leaks therefore positions TDE as the first-line invasive intervention, with TDL reserved for cases where percutaneous access is unsuccessful or the intervention fails. Table 1 summarizes a comparison of the two approaches. However, potential complications of TDE, though infrequent, must be acknowledged, including infection, non-target embolization, glue migration, and, rarely, long-term sequelae of TD occlusion such as peripheral edema, chronic diarrhea, or chylous ascites (22).

**TABLE 1:** Comparative analysis of thoracic duct embolization (TDE) vs. surgical thoracic duct ligation (TDL).

Feature	Thoracic duct embolization (TDE)	Surgical thoracic duct ligation (TDL)	Supporting literature
Invasiveness	Minimally invasive; percutaneous transabdominal access.	Highly invasive; typically requires thoracotomy or VATS.	(20–22)
Anesthesia	Conscious sedation or general anesthesia.	Requires general anesthesia.	(12)
Clinical success rate	∼70–90% (contingent on successful duct cannulation).	High success, often >90%, but typically used after TDE failure.	(13, 20, 23)
Major complications	Non-target embolization, glue migration, post-embolization syndrome (edema, diarrhea).	Surgical site infection, bleeding, persistent air leak, risks of thoracotomy.	(1, 13, 24)
Recovery period	Short; typically 1–2 day hospital stay.	Longer; requires chest drainage and extended hospitalization.	(16, 17)
Role in algorithm	First-line invasive therapy for refractory chyle leaks.	Reserved for TDE failure or when TDE is not feasible.	

This case contributes significantly to the understanding of PLPS by documenting a rare dual presentation of chyloptysis and chylopericardium successfully managed with TDE. It reinforces the necessity of maintaining a high index of suspicion for underlying lymphatic anomalies in patients presenting with unexplained chylous effusions in multiple thoracic compartments. Furthermore, it illustrates the indispensable role of lymphatic imaging in confirming the diagnosis and guiding targeted, minimally invasive therapy, even when the preferred modality (DCMRL) is not viable. The successful outcome achieved with TDE champions its role in the evolving treatment paradigm for PLPS, shifting from expectant or highly invasive approaches toward precise, image-guided lymphatic interventions.

This report is subject to the inherent limitations of a single case study, and the findings may not be universally generalizable.

Future efforts should focus on increasing awareness of PLPS among clinicians across various specialties to facilitate earlier diagnosis. Collaborative, multicenter studies or registries are warranted to better delineate the full clinical spectrum and natural history of PLPS, standardize diagnostic algorithms incorporating advanced imaging, and further refine interventional treatment protocols. Long-term follow-up of patients undergoing embolization for PLPS is also crucial to understand the durability of the intervention and to monitor for potential late recurrences or the development of other lymphatic pathway abnormalities, given the presumed congenital basis of the underlying lymphatic variant.

## Conclusion

This case report demonstrates the successful treatment of PLPS with percutaneous thoracic duct embolization after initial misdiagnosis and ineffective conservative therapy. It highlights the importance of considering PLPS in patients with unexplained chylous effusions and the efficacy of lymphatic intervention as a therapeutic option. Increased awareness and timely diagnosis are essential for optimal management of this rare condition.

## Data Availability

The raw data supporting the conclusions of this article will be made available by the authors, without undue reservation.
